# Risk Factors for Community-Based Reports of Gastrointestinal, Respiratory, and Dermal Symptoms: Findings From a Cohort Study in Australia

**DOI:** 10.2188/jea.JE20130082

**Published:** 2014-01-05

**Authors:** Nusrat Najnin, Andrew Forbes, Martha Sinclair, Karin Leder

**Affiliations:** Department of Epidemiology and Preventive Medicine, School of Public Health and Preventive Medicine, Monash University, Melbourne, Australia

**Keywords:** risk factors, respiratory symptoms, gastrointestinal symptoms, dermal symptoms, swimming, swimming pools, household clustering

## Abstract

**Background:**

Although gastrointestinal (GI), respiratory, and dermal symptoms are common, few studies have conducted concurrent and comparative prospective analyses of risk factors for these 3 morbidity outcomes.

**Methods:**

We used data from a community-based randomized controlled trial among 277 South Australian families to analyze GI (diarrhea, vomiting), respiratory (sore throat, runny nose, cough) and dermal (rash, generalized itch, dermal infection) symptoms.

**Results:**

Log-binomial regression analysis revealed similar risks of GI (adjusted risk ratio [RR], 1.65; 95% CI, 1.05–2.58) and respiratory (RR, 1.68; 95% CI, 1.31–2.15) symptoms among childcare/kindergarten attendees. Swimming in public pools/spas in the current or previous week was associated with all 3 symptom complexes, conferring similar risk for each (RR for GI: 1.33; 95% CI, 0.99–1.77; respiratory: 1.20; 95% CI, 1.04–1.38; dermal: 1.41; 95% CI, 1.08–1.85). Pet ownership was not associated with symptoms. Household clustering of GI and respiratory symptoms was common, and clustering of respiratory symptoms correlated with number of individuals per household.

**Conclusions:**

This simultaneous examination of risk factors for 3 health outcomes yielded new comparative data that are useful for developing prevention strategies.

## INTRODUCTION

Gastrointestinal (GI), respiratory, and dermal diseases are common and cause substantial morbidity and economic loss.^1^^–^^4^ Each of these 3 symptom complexes has a number of underlying causes and can be associated with infection or other (noninfectious) problems such as allergy. Some underlying etiologic causes for these symptoms have known risk factors. For example, previously reported risk factors for GI and respiratory infections include young age, attending an educational institution outside the home, and having another household member who is unwell.^5^^–^^11^ For respiratory infections, factors such as air pollution and smoking are also important.^12^ However, few studies have examined epidemiologic associations for symptomatic episodes of GI, respiratory, or dermal complaints via a prospective, community-based approach, and no previous study has examined risk factors for all 3 morbidity outcomes concurrently.

We attempted to identify risk factors associated with GI, respiratory, and dermal symptoms at the community level among a prospective cohort. Identifying and assessing these risk factors for all 3 disease symptoms from the same cohort within the same time period enables comparison of the strengths of associations and thus provides a new and useful public health perspective.

## METHODS

### Study participants and data collection

As part of a double-blinded, randomized, controlled trial conducted in South Australia from June 2007 to August 2008, weekly diaries were given to 300 families (37% of the total number of households initially approached) to collect health data during a 12-month period. The details of the participant recruitment process are available elsewhere.^13^ Eligibility criteria for inclusion related to the main study goal, which was to determine whether consumption of untreated rainwater contributed to gastroenteritis. The criteria included: using untreated rainwater from an above-ground tank as the usual drinking water source, having at least 4 eligible household members (including at least 2 children aged 1–15 years), home ownership or stable rental history (≥12 months in current home), and having a reasonable command of English. Households were randomly allocated to receive real or sham water treatment devices to treat rainwater for drinking; real devices removed microorganisms from the water, while sham devices did not. Full details on the study and methods used have been reported previously.^14^ In brief, the study families completed a health diary each week, which included reporting of symptoms related to GI, respiratory, and dermal complaints. They also provided exposure information regarding recreational swimming activities, pet ownership, and childcare/school attendance, as well as health-seeking behavior.

### Definitions

We defined a GI symptom as passing a loose stool or vomiting at least once within a 24-hour period. We considered people to have respiratory symptoms if they had a sore throat, runny nose, or cough. A report of rash, generalized itching, or dermal infection was defined as having dermal symptoms. Rather than collect information for each of these symptom complexes daily, we collected information on the overall presence or absence of each type of clinical event during the course of each week.

A recreational swimming setting was defined as a public pool/spa, a private pool/spa, an ocean/beach, or a river/lake/dam. Participants were asked to record whether they had swum during the week and in which setting. No information was recorded regarding duration of swimming, number of times the participant entered the water, or whether the participant had put his/her head underwater.

A cluster of GI, respiratory, and dermal symptoms was defined as development of GI, respiratory, or dermal symptoms, respectively, in more than 1 household member in the same or consecutive weeks. Each cluster was considered to have ended if 2 weeks elapsed with no symptoms reported by any household member. Participants could appear in more than 1 cluster over the period of observation. Sporadic GI, respiratory, and dermal symptoms were defined as cases that occurred outside of a cluster.

### Data management

Completed health diaries were mailed to the study center (Monash University) every 4 weeks. Diaries were scanned, and the accuracy and completeness of data were verified using Cardiff Teleform software (version 10.1, 2006; Vista, CA, USA) before data entry into a Microsoft Access database. Reporting participants were telephoned for clarification if information was missing or ambiguous.

### Data analysis

The number of weeks with valid information was determined for each of the 3 symptom complexes and for information on swimming exposure. Analyses of the effect of swimming in different settings during the current or previous week on incident events in relation to the 3 symptom complexes during the current week were performed using log-binomial regression to estimate risk ratios (RRs),^15^ accounting for family clustering using robust standard errors and adjusting for age, sex, season, and swimming in different settings. To estimate incident rather than prevalent events, the analyses were restricted to weeks when each individual did not experience the symptom complex of interest in the prior week. Associations of risk factors with being in a symptom cluster versus a sporadic event or no event were also estimated using log-binomial regression accounting for family clustering. Each independent variable was evaluated for confounding and effect modification. Two-sided *P* values less than 0.05 were considered statistically significant. All calculations were performed using Stata version 11.1.^16^

### Ethical considerations

During enrollment, written informed consent was obtained from all adult household members and from parents and guardians on behalf of children. This study received approval from the Monash University Standing Committee on Ethics in Research Involving Humans (SCERH; 2006/555EA) and the South Australia Department of Health Human Research Ethics Committee.

## RESULTS

The original study comprised 300 households with 1352 residents. We excluded 23 households who failed to return any health diaries (21 households) or had missing demographic information (2 households). Ultimately, our analysis included 277 households comprising 1237 participants. The households who failed to return health diaries and were therefore excluded from the analysis and the households that were included in the analysis had similar demographic characteristics. A comparison of gastroenteritis rates between groups with real or sham water treatment devices showed no significant difference, indicating that drinking untreated rainwater did not contribute appreciably to health outcomes.^14^ Therefore, results from both sham and real filter groups were combined, and this cohort was considered generally representative of households with young children.

The mean age of the study participants was 24.1 years (age range: 0.6–78.6 years); 11% (*n* = 132) of the study participants were children aged 5 years or younger. The numbers of male and female participants in the study were similar (Table 1). Among the total of 1237 study participants, 54% (*n* = 674) were attending an educational institution (child care/kindergarten, primary/secondary school, university) at the time of the study.

**Table 1.** Demographic characteristics of study participants, Adelaide, Australia (June 2007–August 2008)

Characteristics	*n* = 1237 (%)
**Age**	
≤5 years	132 (11)
>5 to ≤15 years	489 (40)
>15 years	616 (50)
**Sex**	
Male	626 (51)
Female	611 (49)
**Educational status**	
**Currently attending an educational institution**	
Attending childcare/preschool	98 (8)
Primary	406 (33)
Secondary	139 (11)
College/university	31 (3)
**Education completed^a^**	
Primary	7 (1)
Secondary/commercial/technical	250 (20)
College/university	273 (22)

^a^Data missing for 38 (3%) participants.

Children attending child care/kindergarten were at higher risk of GI (RR, 1.65; 95% CI, 1.05–2.58) and respiratory symptoms (RR, 1.68; 95% CI, 1.31–2.15) as compared with all others (Table 2). The risk of reporting dermal symptoms showed no such association. The similarity in the strength of the association of childcare/kindergarten attendance with GI and respiratory symptoms suggests that such attendance is an equally strong risk factor for these health outcomes.

**Table 2.** Association of attendance at childcare, school, or other educational institution, pet ownership, and swimming exposure during the previous or current week with GI, respiratory, and dermal symptoms, Adelaide, Australia (June 2007–August 2008^a^) (*n* = 1237)

Attending institution outside home	Unadjusted		Adjusted^b^		
	
	Risk ratio	95% CI	Risk ratio	95% CI	*P* value
**GI**					
Attending child care/kindergarten^c^	2.51	2.02, 3.12	1.65	1.05, 2.58	
Having a pet at home^d^	0.98	0.68, 1.41	—	—	
Swimming in any setting	1.24	0.98, 1.56	1.19	0.94, 1.52	0.149
Swimming in public pool/spa^e^	1.5	1.15, 2.04	1.33	0.99, 1.77	0.057
Swimming in private pool/spa^e^	0.75	0.55, 1.03	0.83	0.60, 1.15	0.251
Swimming in ocean and/or river^e^	0.94	0.68, 1.31	0.99	0.70, 1.39	0.948
**Respiratory**					
Attending child care/kindergarten^c^	2.52	2.09, 3.03	1.68	1.31, 2.15	
Having a pet at home^d^	1.11	0.83, 1.47	—	—	
Swimming in any setting	1.06	0.92, 1.22	1.09	0.97, 1.23	0.153
Swimming in public pool/spa^e^	1.34	1.13, 1.58	1.20	1.04, 1.38	0.014
Swimming in private pool/spa^e^	0.71	0.58, 0.87	0.98	0.80, 1.20	0.842
Swimming in ocean and/or river^e^	0.77	0.62, 0.95	0.92	0.72, 1.18	0.517
**Dermal**					
Attending child care/kindergarten^c^	2.74	1.71, 4.39	0.96	0.52, 1.77	
Having a pet at home^d^	1.45	0.81, 2.60	—		—
Swimming in any setting	1.82	1.43, 2.33	1.59	1.26, 2.01	<0.001
Swimming in public pool/spa^e^	2.07	1.50, 2.87	1.41	1.08, 1.85	0.013
Swimming in private pool/spa^e^	1.16	0.74, 1.80	1.16	0.77, 1.74	0.471
Swimming in ocean and/or river^e^	1.08	0.76, 1.52	1.42	0.95, 2.14	0.088

Abbreviation: GI, gastrointestinal.

^a^All analysis accounted for clustering by household.

^b^Risk ratios adjusted for age, sex, season, and household clustering.

^c^Comparator group: Attending primary school or a higher educational institution or not attending any educational institute.

^d^Dogs, cats, birds, and fish.

^e^Adjusted for swimming in other settings; comparator group: swimming in other settings and non-swimmers.

Overall, 77% (*n* = 957) of the study participants reported swimming at least once during the study period. There was no difference in swimming status between males and females (51% vs 49%; *P*: 0.22). Among those who reported having swum at least once, many swam in more than 1 setting. Overall, 75% (*n* = 722) swam in a public pool or spa, 62% (*n* = 591) swam in a private pool or spa, 56% (*n* = 538) swam in the ocean, and 22% (*n* = 210) swam in a river, dam, or lake. The largest proportion of swimmers were those aged 5 to 15 years (45%; *n* = 433). Fewer people swam during winter as compared with the other seasons.

Among those who swam at least once, swimming in a public pool/spa during the current and/or previous week was significantly associated with all 3 symptom complexes, as compared with swimming in other settings and not swimming, in univariate regression analysis. These associations remained significant after adjusting for potential confounders (Table 2). The strengths of the associations of public pool/spa exposure with dermal (RR, 1.41; 95% CI, 1.08–1.85), GI (RR, 1.33; 95% CI, 0.99–1.77), and respiratory symptoms (RR, 1.2; 95% CI, 1.04–1.38) were similar. In multivariable analysis, swimming in any setting was associated only with dermal symptoms. We found no significant association of swimming exposure in a private pool/spa or in an ocean/river/lake/dam with any disease symptoms of interest (Table 2). Moreover, if we restricted the multivariable regression analysis to swimming in the previous week only, only swimming in a public pool and respiratory symptoms were significantly associated (*P* = 0.022). Corresponding analysis restricted to swimming and having symptoms during the same week showed that dermal symptoms were associated with swimming in any setting (*P* = 0.003) and swimming in a public pool/spa (*P* = 0.029), and that GI symptoms were associated with swimming in a private pool/spa (*P* = 0.018). We found no association of GI, respiratory, or dermal symptoms with pet (cat/dog/fish/bird) ownership (Table 2).

Of the 45% (*n* = 561) of participants who had GI symptoms during the study period, 63% (*n* = 355) were part of a cluster and 37% (*n* = 211) were sporadic cases. There was a total of 287 GI symptom clusters, distributed among 124 (45%) of households. The mean number of GI clusters per household in those recording at least 1 cluster was 2.3 (median 2, maximum 15), and the mean number of symptomatic weeks among individuals in a GI cluster(s) was greater than that among those who were never part of a cluster (2.5 vs 1.5, *P* < 0.001). We found no correlation between number of members per household and number of study participants in a GI cluster (*P* = 0.11). Those in at least 1 GI cluster were younger (mean 20.3, median 11.4 years) than those not in a GI cluster (mean 25.7, median 16.6 years, *P* < 0.001).

Overall, 80% (*n* = 987) of study participants had respiratory symptoms, among whom 94% (*n* = 929) were part of a cluster and 6% (*n* = 84) were sporadic cases. The 1568 respiratory symptom clusters reported involved 240 households (87%). People in a respiratory cluster(s) reported more weeks with symptoms than did sporadic cases (mean 4.8 vs 1.8, *P* < 0.001). The mean number of respiratory clusters per household was 6.5 (median 5, maximum 33), and there was a correlation between number of household members and total number of participants involved a cluster (*P* < 0.001). The mean ages for those in (22.9 years) and outside (27.9 years) a cluster were different (*P* < 0.001).

Of the 31% (*n* = 273) of study participants who had dermal symptoms during the study, 39% (*n* = 107) were part of a cluster and 61% (*n* = 166) were sporadic cases. There were 107 dermal symptom clusters affecting 39 (14%) of households. More symptomatic weeks were reported by those in a dermal cluster(s) (mean, 5.8 weeks vs 3.4 for sporadic cases, *P* = 0.012). The maximum number of dermal clusters per household was 28 (mean 4.7, median 2). There was no correlation between number of household members and number of people in a cluster (*P* = 0.124). The mean age for individuals in a cluster (17.3 years) was lower than that for those not in a cluster (24.8 years) (*P* < 0.001).

For all 3 symptom complexes, the identified risk factors for being part of a household cluster were age under 5 years and attendance at a child care/kindergarten (Tables 3, 4 and 5).

**Table 3.** Demographics of individuals within and outside a GI symptom cluster, Adelaide, Australia (June 2007–August 2008^a^) (*n* = 1235)

Demographic characteristics	People in cluster *n* = 355 (%)	People with sporadic GI symptoms *n* = 211 (%)	People with no GI symptoms *n* = 669 (%)	Risk ratio^b^ (95% CI)	*P* value
**Sex**					
Male	169 (48)	104 (50)	352 (53)	1.00	
Female	186 (52)	107 (51)	317 (47)	1.13 (0.96, 1.32)	0.131
**Age**					
<5 years	78 (22)	22 (10)	32 (5)	2.49 (2.01, 3.05)	<0.001
5 to 15 years	131 (37)	87 (41)	270 (40)	1.13 (0.93, 1.37)	0.208
>15 years	146 (41)	102 (48)	367 (55)	1.00	—
**Attending educational institution**					
Attending child care/kindergarten	60 (17)	15 (7)	23 (3)	2.32 (1.89, 2.86)	<0.001
Attending primary school or higher educational institution	147 (41)	100 (47)	328 (49)	0.97 (0.80, 1.18)	0.76
Not attending any educational institution	148 (42)	96 (46)	318 (48)	1.00	—

Abbreviation: GI, gastrointestinal.

^a^Cluster was defined as >1 person in a household having GI symptoms during the current or previous week.

^b^Risk ratio for being in a cluster vs not being in cluster (people with sporadic and no GI symptoms combined), using binary regression adjusted for clustered family design.

**Table 4.** Demographics of individuals within and outside a respiratory symptom cluster, Adelaide, Australia (June 2007–August 2008^a^) (*n* = 1235)

Demographic characteristics	People in cluster *n* = 929 (%)	People with sporadic respiratory symptoms *n* = 84 (%)	People with no respiratory symptoms *n* = 220 (%)	Risk ratio^b^ (95% CI)	*P* value
**Sex**					
Male	465 (50)	39 (46)	121 (55)	1.00	—
Female	464 (50)	45 (54)	101 (46)	1.02 (0.96, 1.10)	0.457
**Age**					
<5 years	118 (13)	7 (8)	7 (3)	1.24 (1.15, 1.34)	<0.001
5 to 15 years	369 (40)	38 (45)	82 (37)	1.05 (0.99, 1.11)	0.102
>15 years	442 (48)	39 (46)	1433 (60)	1.00	—
**Attending educational institution**					
Child care/kindergarten	87 (9)	7 (8)	4 (2)	1.21 (1.11, 1.32)	<0.001
Attending primary school or higher educational institution	432 (47)	44 (52)	100 (45)	1.03 (0.97, 1.09)	0.84
Not attending any educational institution	410 (44)	33 (39)	118 (53)	1.00	—

^a^Cluster was defined as >1 person in a household having respiratory symptoms during the current or previous week.

^b^Risk ratios for being in a cluster vs not being in cluster (people with sporadic and no respiratory symptoms combined), using binary regression adjusted for clustered family design.

**Table 5.** Demographics of the individuals within and outside a dermal symptom cluster, Adelaide, Australia (June 2007–August 2008^a^) (*n* = 1235)

Demographic characteristics	People in cluster *n* = 107 (%)	People with sporadic dermal symptoms *n* = 166 (%)	People with no dermal symptoms *n* = 962 (%)	Risk ratio^b^ (95% CI)	*P* value
**Sex**					
Male	49 (46)	76 (46)	499 (52)	1.00	—
Female	58 (54)	90 (54)	463 (48)	1.21 (0.83, 1.77)	0.33
**Age**					
<5 years	24 (22)	34 (21)	74 (8)	3.39 (2.04, 5.62)	<0.001
5 to 15 years	50 (47)	74 (45)	364 (38)	1.91 (1.31, 2.77)	0.001
>15 years	33 (31)	58 (35)	524 (55)	1.00	—
**Attending educational institution**					
Child care/kindergarten	18 (17)	28 (17)	52 (5)	2.37 (1.68, 4.62)	0.002
Attending primary school or higher educational institution	52 (49)	83 (50)	440 (46)	1.37 (0.93, 2.02)	0.11
Not attending any educational institution	37 (35)	55 (33)	470 (49)	1.00	—

^a^Cluster was defined as >1 person in a household having dermal symptoms during the current or previous week.

^b^Risk ratios for being in a cluster vs not being in cluster (people with sporadic and no dermal symptoms combined) using binary regression, adjusted for clustered family design.

## DISCUSSION

To our knowledge, this is the first prospective longitudinal cohort study to examine risk factors associated with community reports of GI, respiratory, and dermal symptoms concurrently. Among our study participants, those who attended childcare or kindergarten were more likely to suffer from respiratory symptoms, even after adjustment for age. People swimming in public pools or spas had an increased risk of reporting all 3 symptom complexes, and household clusters of GI and respiratory symptoms were common.

Attending childcare or kindergarten was previously reported as a risk factor for GI and respiratory symptoms.^5^^,^^17^^–^^20^ Our results support those findings but additionally suggest that childcare/kindergarten attendance increases the risks of these health outcomes by approximately 60%. One likely reason for the vulnerability to illness among young children attending childcare is close contact with other infected children (and/or staff) in a crowded environment, particularly as hygiene measures may be compromised in this setting. We observed no strong association between dermal symptoms and attendance at educational institutions, consistent with the premise that transmission of contagious dermal symptoms is less likely. We also found that keeping any kind of pet at home was not a risk factor for any of the 3 symptoms of interest. This is supported by studies conducted in other settings.^21^^–^^25^

Recreational swimming is another recognized risk factor for GI, respiratory, and dermal symptoms,^26^^–^^28^ although no previous study examined whether there is a differential impact in relation to type of swim setting for all 3 symptom complexes. We found that recreational swimming in any body of water was significantly associated only with dermal symptoms. However, swimming in a public pool/spa was an identified risk factor for GI, respiratory, and dermal symptoms (adjusted RRs for all 3 symptom complexes, 1.2–1.4). Outbreaks of gastroenteritis associated with swimming in a public pool or spa are reported frequently,^29^^,^^30^ but the relationship between sporadic gastroenteritis and swimming is complex, as it reflects factors such as the background pathogen load in the source water, the likelihood of water contamination due to fecal pathogen excretion by other swimmers,^31^ the impact of any disinfection procedures (eg, chlorination) on pathogen concentration,^32^ and the volume of water ingested by the swimmer.

While it is important to treat pool water with disinfectants to kill microorganisms and reduce the chance of disease, these chemical products and/or their by-products might contribute to respiratory and dermal symptoms by inducing an allergic reaction.^28^^,^^33^^,^^34^ However, swimming in a private pool, which would also involve chemical disinfectants (albeit at potentially lower concentrations), was not significantly associated with symptoms.

The final aspect examined was the frequency of clustered symptoms among household members. Although presence of concurrent symptoms among householders was reported previously,^8^^–^^10^^,^^35^ many of the relevant studies were performed in the setting of a community outbreak or as a follow-up of a laboratory-confirmed case of an individual pathogen^36^^–^^39^ rather than in a prospective community-based study. In the present study, clusters of dermal symptoms affected 31% of households, as compared with 45% and 80% for GI and respiratory symptoms, respectively. For all 3 symptom types, being in at least 1 cluster was associated with a higher mean number of weeks with symptoms, as compared with sporadic cases. The 2 demographic characteristics most strongly associated with being in any type of cluster were age younger than 5 years and attending childcare or kindergarten, which confirms the findings of previous research conducted in a variety of settings.^6^^,^^7^^,^^39^^–^^42^ Respiratory symptoms were most common overall, and clustering of respiratory symptoms was also more common in larger households. These findings may reflect the comparative transmissibility of respiratory, GI, and dermal pathogens.

In contrast to earlier reports of family clustering of GI and respiratory symptoms, which gathered evidence from known outbreaks or laboratory surveillance data, our prospectively collected data are more likely to reflect levels of community-based clustering. However, we were not able to examine the underlying reasons for clustering of symptoms within families, which could be due to common exposure, secondary spread, simultaneous occurrence of unrelated sporadic cases, or (for noninfectious etiologies) familial sensitivity. For example, clustered dermal symptoms do not necessarily reflect pathogen transmission and may be due to atopy within families.

Our study has several limitations. First, we relied on self-reported data over a 1-year time period. It is possible that response fatigue may have meant some people did not report all symptoms, which may have resulted in under-reporting and therefore underestimation of the strength of association for some risk factors. Second, we collected information on the presence or absence of different symptoms using broad symptom-based case definitions. Therefore, the results must be interpreted with caution, as not all symptoms were necessarily serious or infectious. Nevertheless, they are indicative of the frequency and burden of each of the 3 symptom types in the community. Third, our case definition of swimming considered swimming during the current and/or previous week and GI, respiratory, and dermal symptoms during the current week. Consideration of the current week in our case definition means that we measured exposure (swimming) and outcome in the same week; thus, we cannot be entirely certain that exposure preceded outcome. Additionally, we measured weeks, rather than days, with symptoms and therefore cannot precisely define the start and end points of episodes. Finally, our findings may not be generalizable across whole communities. We deliberately enrolled selected English-speaking households in South Australia with at least 2 children aged 1 to 15 years. Therefore, our results reflect the demographics of those included, namely, young families living in urban Adelaide. While the results may thus not be applicable to all other populations, they are nevertheless likely to be relevant for families in urban areas of developed countries.

In summary, in a prospective cohort of 277 Australian families, we confirmed and extended previous reports of risk factors for illness by performing a prospective community-based study that simultaneously examined respiratory, GI, and dermal health complaints. Attendance at childcare or kindergarten was similarly associated with GI and respiratory symptoms. Recreational swimming in public pools was an equally strong risk factor for GI, respiratory, and dermal symptoms. Clustering of symptoms within households was common for GI and respiratory symptoms, although more respiratory clusters were seen. Prospectively assessing risk factors for 3 symptom complexes together in 1 cohort during 1 time period is new and enabled us to compare risk ratios and strengths of associations for different risk factors. These comparative data are helpful in prioritizing prevention strategies for various health outcomes.
